# ﻿A new species of forest hedgehog (*Mesechinus*, Erinaceidae, Eulipotyphla, Mammalia) from eastern China

**DOI:** 10.3897/zookeys.1185.111615

**Published:** 2023-11-28

**Authors:** Zifan Shi, Hongfeng Yao, Kai He, Weipeng Bai, Jiajun Zhou, Jingyi Fan, Weiting Su, Wenhui Nie, Shuzhen Yang, Kenneth O. Onditi, Xuelong Jiang, Zhongzheng Chen

**Affiliations:** 1 Collaborative Innovation Center of Recovery and Reconstruction of Degraded Ecosystem in Wanjiang Basin Co-founded by Anhui Province and Ministry of Education, School of Ecology and Environment, Anhui Normal University, Wuhu, China Anhui Normal University Wuhu China; 2 Key Laboratory of Conservation and Application in Biodiversity of South China, School of Life Sciences, Guangzhou University, Guangzhou, China Guangzhou University Guangzhou China; 3 Institute of Nihewan Archaeology, College of History and Culture, Hebei Normal University, Shijiazhuang, China Hebei Normal University Shijiazhuang China; 4 Zhejiang Forest Resources Monitoring Center, Hangzhou, China Zhejiang Forest Resources Monitoring Center Hangzhou China; 5 State Key Laboratory of Genetic Resources and Evolution & Yunnan Key Laboratory of Biodiversity and Ecological Security of Gaoligong Mountain, Kunming Institute of Zoology, Chinese Academy of Sciences, Kunming, China Institute of Zoology, Chinese Academy of Sciences Kunming China; 6 Management Office, National Nature Reserve of Mount Tianmu, Hangzhou, China National Nature Reserve of Mount Tianmu Hangzhou China

**Keywords:** Anhui, mammals, phylogeny, taxonomy

## Abstract

The hedgehog genus *Mesechinus* (Erinaceidae, Eulipotyphla) is currently comprised of four species, *M.dauuricus*, *M.hughi*, *M.miodon*, and *M.wangi*. Except for *M.wangi*, which is found in southwestern China, the other three species are mainly distributed in northern China and adjacent Mongolia and Russia. From 2018 to 2023, we collected seven *Mesechinus* specimens from Anhui and Zhejiang provinces, eastern China. Here, we evaluate the taxonomic and phylogenetic status of these specimens by integrating molecular, morphometric, and karyotypic approaches. Our results indicate that the Anhui and Zhejiang specimens are distinct from the four previously recognized species and are a new species. We formally described it here as *Mesechinusorientalis***sp. nov.** It is the only *Mesechinus* species occurring in eastern China and is geographically distant from all known congeners. Morphologically, the new species is most similar to *M.hughi*, but it is distinguishable from that species by the combination of its smaller size, shorter spines, and several cranial characteristics. *Mesechinusorientalis* sp. nov. is a sister to the lineage composed of *M.hughi* and *M.wangi* from which it diverged approximately 1.10 Ma.

## ﻿Introduction

In recent years, interest in the faunal inventory of insectivorous mammals in different countries has increased (Kryštufek and Motokawa 2018; Andreychev and Kuznetsov 2020). The forest hedgehog genus *Mesechinus* Ognev, 1951 is one of five extant genera in the subfamily Erinaceinae. *Mesechinus* was previously regarded as a subgenus of *Erinaceus* Linnaeus, 1758 or *Hemiechinus* Fitzinger, 1866 (Pavlinov and Rossolimo 1987; Corbet 1988; Bannikova et al. 2002). Frost et al. (1991) promoted it to full-genus status, a conclusion supported by analysis of morphological characters (Gould 1995) and chromosomal data (Korablev et al. 1996). The most distinctive morphological character distinguishing *Mesechinus* from *Erinaceus* and *Hemiechinus* is the unique shape of the suprameatal fossa; the lateral border of this fossa is somewhat U-shaped in *Mesechinus* but C-shaped in the other erinaceine genera (Frost et al. 1991).

Currently, four species are recognized in the genus, including *M.dauuricus* (Sundevall, 1842), *M.hughi* (Thomas, 1908), *M.miodon* (Thomas, 1908), and *M.wangi* He, Jiang & Ai, 2018 (Wilson and Mittermeier 2018). *Mesechinus* species mainly occur in northern China and adjacent Mongolia and Russia, with an isolated species (*M.wangi*) on Mount Gaoligong, Yunnan, southwestern China (Frost et al. 1991; Ai et al. 2018). *Mesechinusdauuricus* and *M.hughi* mainly inhabit semidesert habitats, including cold-temperate deciduous and temperate deserts, warm-temperate deserts, grasslands and deciduous broad-leaf forests, *M.miodon* mainly inhabits semiarid and dry steppe habitats and subalpine and low-elevation coniferous forests, and only *M.wangi* inhabits tropical or subtropical rainforest (Ai et al. 2018; Wilson and Mittermeier 2018).

**Figure 1.**
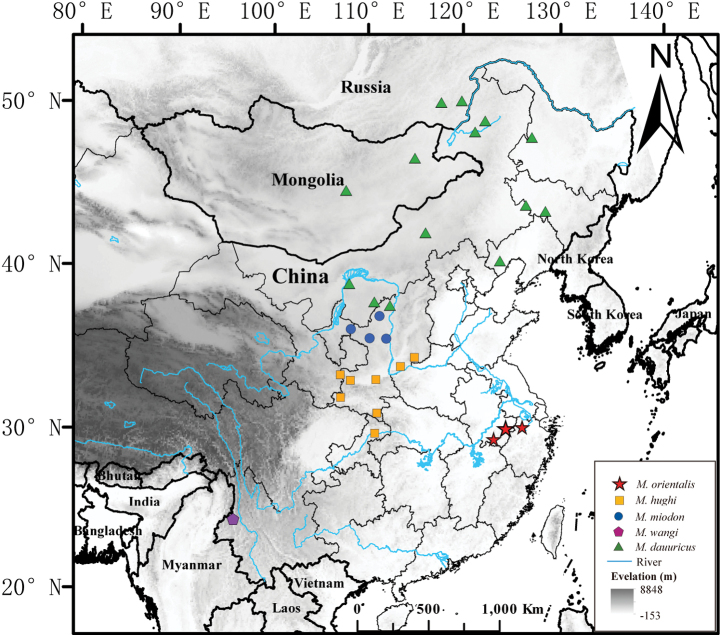
Distribution of the genus *Mesechinus*.

Hugh’s Hedgehog (*M.hughi*) is the smallest species of *Mesechinus* and is mainly distributed in southern Shaanxi, southern Shanxi, and northern Sichuan in China (Ai et al. 2018) (Fig. 1). This dark-coloured hedgehog with no all-white spines was first described by Thomas (1908) based on specimens from Paochi (= Baoji), Shaanxi, China. Chen et al. (2020) reported the first record of *M.hughi* in eastern China based on a specimen collected from Xuancheng, Anhui Province. They pointed out that Anhui *Mesechinus* specimen was genetically distant from Shaanxi specimens (4.9–5.3% of CYT B gene) and may have undergone isolated differentiation (Chen et al. 2020). Recently, we obtained a CYT B sequence of *M.wangi*. Our preliminary phylogenetic analysis revealed that the Anhui specimens form a lineage sister to the lineage composed of *M.hughi* from Shaanxi and *M.wangi*, which suggests that additional studies with more specimens were necessary to confirm the taxonomic status of the Anhui *Mesechinus*.

**Figure 2.**
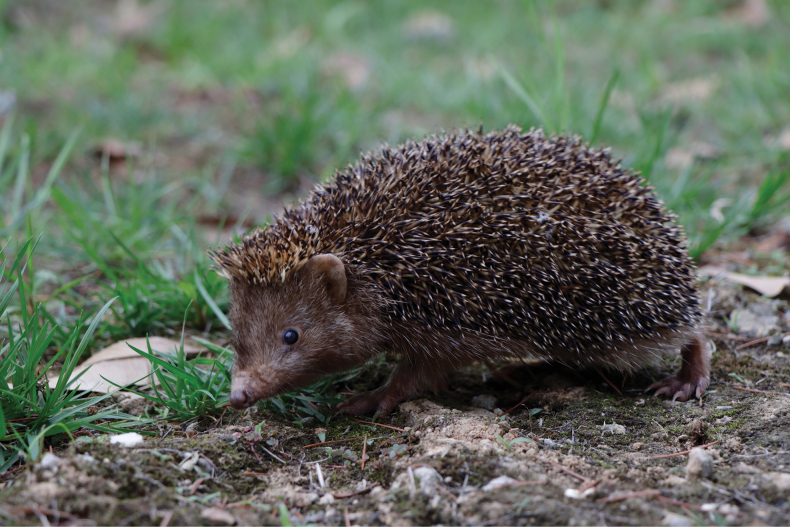
A living *Mesechinusorientalis* sp. nov. (XC 2205003) from Xuancheng, Anhui.

From 2022 to 2023, we collected six specimens of *Mesechinus* from Anhui and Zhejiang provinces, eastern China (Fig. 2). Our morphological and molecular results reveal the eastern China samples differ from *M.hughi* and other known *Mesechinus* species. We recognize it as a new species, *Mesechinusorientalis* sp. nov., which we describe here.

## ﻿Materials and methods

### ﻿Sampling

Seven *Mesechinusorientalis* sp. nov. specimens, including a specimen collected by Chen et al. (2020), were obtained from Anhui and Zhejiang provinces in eastern China (Suppl. material 1). Our specimens were euthanized, and liver or muscle tissues were extracted and preserved in pure ethanol. Skulls were extracted and cleaned. All the specimens and tissues were deposited at Anhui Normal University (AHNU). Animals were handled consistent with the Guidelines of the American Society of Mammalogists (Sikes 2016).

### ﻿Morphological measurement and analysis

Five external measurements, including weight (W), head–body length (HB), tail length (TL), hind-foot length (HF), and ear length (EL) of *M.orientalis* sp. nov. were measured in the field to the nearest 1 g or 1 mm. Twelve craniodental variables were measured using digital calipers graduated to 0.01 mm following Pan et al. (2007) and Ai et al. (2018): greatest length of the skull (GLS), condylobasal length (CBL), basal length (BL), cranial height (CH), palatal length (PL), zygomatic breadth (ZMB), interorbital breadth (IOB), mastoid width (MTW), greatest width of nasal (GWN), breadth of first upper molar (BM1), length of upper tooth row (LUTR), and length of below tooth row (LBTR). Comparative morphological data of other *Mesechinus* species were obtained from Ai et al. (2018), which included 4 *M.wangi*, 18 *M.miodon*, 31 *M.hughi*, and 13 *M.dauuricus*.

Thirty-seven complete skulls were used for PCA, including specimens of 3 *M.wangi*, 20 *M.hughi*, 6 *M.miodon*, 1 *M.dauuricus*, and 7 *M.orientalis* sp. nov. Morphometric variation was analyzed using a principal component analysis (PCA) in SPSS 19.0 based on 12 log_10_-transformed cranial measurements. To further confirm the validity of the potential new species, we coded the characters of *Mesechinus* species according to Gould (1995). In this procedure, we systematically compared the morphological characteristics of the new species with other *Mesechinus* species, especially the most morphologically similar species, *M.hughi*.

### ﻿Mitogenome sequencing, assembly, and annotation

We used next-generation sequencing (NGS) to obtain the complete mitochondrial genome of *M.orientalis* sp. nov. Illumina high-throughput sequencing platform was employed for sequencing with a strategy of 150 paired-ends, and the quality was checked using FastQC (de Sena Brandine and Smith 2021). The mitochondrial genome assembly was performed using NOVOPlasty (Dierckxsens et al. 2017).

The mitochondrial genome was annotated using MitoZ in the MITOS WebServer with analytical parameters set using the vertebrate genetic code (Bernt et al. 2013; Meng et al. 2019). Geneious v. 9.0.2 (Kearse et al. 2012) was used to examine all mitochondrial genes. The obtained sequences were edited and aligned with MEGA v. 11(Tamura et al. 2021). The newly obtained mitogenome has been deposited in GenBank (accession number OR774964).

### ﻿Phylogenetic analysis

The mitochondrial genomes of four other *Mesechinus* species, and six erinaceid species, including representatives of *Paraechinus* Trouessart, 1879, *Hemiechinus*, and *Atelerix* Pomel, 1848, were downloaded from GenBank and included in our analyses. Mitochondrial genomes of *Neotetracussinensis* Trouessart, 1909 and *Hylomyssuillus* Müller, 1840, also obtained from GenBank, were used as the outgroup (Table 1). The phylogenetic analyses were conducted using the two rRNA and 12 coding genes on the heavy chain and excluded ND6 on the light chain. Each gene was aligned using MUSCLE and then checked manually.

**Table 1.** Samples used for molecular phylogenetic analysis in this study.

Subfamily	Species	Museum code	Collection localities	GenBank no.
Galericinae	*Hylomyssuillus*		Java, Indonesia	AM905041
	*Neotetracussinensis*		Pingshan, Yibin, Sichuan, China	NC_019626
Erinaceinae	*Paraechinusmicropus*	USNM369316		OP654708
	*Hemiechinusauritus*			AB099481
	*Atelerixalbiventris*	USNM325883		OP654703
	*Erinaceusamurensis*		Gongwon, Korea	KX964606
	*Mesechinusmiodon*		Yulin, Shaanxi, China	KT824773
	*M.dauuricus*	KIZ200908002		OP654710
	*M.wangi*	GLGS0907001		OP654712
	*M.hughi*	KIZ200908004		OP654727
	*M.orientalis* sp. nov.	XC 2205003	Xuancheng, Anhui, China	OR774964

To reconstruct the phylogenetic relationships, maximum-likelihood (ML) and Bayesian-inference (BI) analyses were performed in IQ-TREE and MrBayes, respectively, in PhyloSuite (Zhang et al. 2020). The phylogenetic tree was visualized and annotated in tvBOT (Xie et al. 2023). The best-fit partitioning schemes were estimated based on the Bayesian Information Criterion (BIC) using PartitionFinder 2 (Lanfear et al. 2017).

### ﻿Divergence time estimation

BEAST v. 2.6 (Bouckaert et al. 2014) was used to estimate divergence times in the CIPRES Science Gateway (Miller et al. 2015). The data were partitioned according to the results of PartitionFinder 2 (Suppl. material 2). We used the unlinked site model and linked clock model and time tree across partitions, and the relaxed lognormal clock model and a birth-death model for the tree prior. Two secondary calibrations were used: (1) the most recent common ancestor of the subfamilies Galericine and Erinaceinae, which was estimated at ca 28.3–48.8 Ma (Meredith et al. 2011) using a lognormal distribution prior (mean = 3.61, SD = 0.142, offset = 0); and (2) The most recent common ancestor of Erinaceinae, which was estimated at ca 6.97 Ma (He et al. 2021), with a normal distribution prior (mean = 6.97, sigma = 2.05, offset = 0). The analyses were conducted twice, each for 100 million generations, sampling every 10000 generations. The posterior distribution of each parameter in the log file was estimated using Tracer v. 1.7 (Rambaut et al. 2018) to ensure that the effective sampling size of all parameters was greater than 200. For all BEAST analyses, we compiled time trees with TreeAnnotator v. 2.6 (Bouckaert et al. 2014) and excluded 10% of each chain as burn-in. The generated tree was viewed in FigTree v. 1.4 (Rambaut 2017) and beautified in tvBOT (Xie et al. 2023).

### ﻿Cell culture and karyotype analysis

A female individual of *M.orientalis* sp. nov. (XC 2205003) collected in May 2022 was used for cell cultures. Standard procedures were applied for fibroblast culture, chromosome preparation, and G-banding. Two fibroblast cell lines derived from *M.orientalis* sp. nov. (XC 2205003) were established and deposited in the Kunming Cell Bank, Yunnan, China. A CytoVision system (Applied Imaging Co., USA) with a CCD camera mounted on a Zeiss microscope (Germany) was used to karyotype analysis. Chromosomes of *M.orientalis* sp. nov. (XC 2205003) were numbered according to *M.wangi* (Ai et al. 2018).

## ﻿Results

### ﻿Morphological analyses

Summaries of external morphology and craniodental measurements are given in Table 2. According to the measurements, *Mesechinusorientalis* sp. nov. (HB = 188.83 mm ± 8.13; GLS = 49.95 mm ± 1.69) is similar in size to *M.hughi* (HB = 189.71 mm ± 23.80; GLS = 49.39 mm ± 1.54) but much smaller than *M.wangi*, *M.dauuricus*, and *M.miodon* (Table 2).

**Table 2.** External and cranial measurements (mm) of *Mesechinus* specimens examined; mean ± S), range for each measurement, and number of specimens measured (*n*) are given.

	***M.orientalis* sp. nov.**	***M.hughi***	***M.dauuricus***	***M.miodon***	***M.wangi***
	***n* = 7**	***n* = 31**	***n* = 13**	***n* = 18**	***n* = 4**
W	339 ± 52.97	341 ± 125.75	562 ± 124.31	505 ± 154.03	401 ± 43.27
	299–414; 3	112–750; 31	423–840; 11	230–750; 6	336–449; 4
HB	188.83 ± 8.13	189.71 ± 23.80	373.91 ± 21.35	205 ± 23.53	208.75 ± 21.90
	176–198; 6	148–232; 31	175–261; 12	120–220; 17	180–140; 4
TL	23.50 ± 3.77	19.23 ± 3.26	24.08 ± 3.50	33.22 ± 5.07	17.08 ± 1.78
	16–27; 6	12–24; 27	17–30; 12	25–43; 17	14–18; 4
HF	36.75 ± 3.19	37.97 ± 4.29	34.74 ± 7.08	58.80 ± 82.43	47.00 ± 1.12
	31–40; 6	30–47; 31	18–41; 12	35–38; 16	45–48; 4
EL	26.00 ± 2.66	22.94 ± 3.93	31.19 ± 3.28	28.81 ± 3.03	30.00 ± 1.49
	23–30; 6	16–33; 31	22–34; 11	24–35; 17	28–31; 4
GLS	49.95 ± 1.69	49.39 ± 1.54	55.18 ± 3.07	54.10 ± 2.10	54.75 ± 0.70
	47.64–51.76; 7	45.10–52.40; 23	50.20–58.40; 12	49.30–57.20; 14	53.70–55.60; 4
CBL	49.49 ± 1.64	48.46 ± 1.58	54.72 ± 2.83	53.18 ± 2.35	54.55 ± 0.59
	47.27–51.42; 7	44.40–51.20; 23	49.40–57.40; 13	48.50–56.30; 11	53.60–55.20; 4
CH	15.42 ± 0.54	16.14 ± 0.95	17.76 ± 2.00	18.67 ± 0.66	17.13 ± 0.60
	14.46–16.39; 7	14.90–18.20; 21	17.20–19.10; 9	17.80–19.70; 6	16.10–17.60; 4
BL	46.66 ± 1.45	45.55 ± 1.29	51.83 ± 1.94	49.64 ± 2.04	50.00 ± 1.37
	44.47–48.28; 7	43.20–48.80; 21	48.10–54.50; 13	44.70–52.30; 14	47.70–51.30; 4
PL	27.46 ± 0.77	26.58 ± 0.62	28.60; 1	28.82 ± 1.41	30.25 ± 0.50
	26.17–28.52; 7	25.70–28.40; 21		27.00–32.18; 14	29.50–30.80; 4
ZMB	29.62 ± 1.51	28.90 ± 1.68	32.62 ± 2.82	32.77 ± 2.09	33.97 ± 0.19
	27.78–31.41; 7	25.70–32.00; 22	28.40–36.40; 13	28.70–37.08; 14	33.70–34.10; 3
IOB	12.29 ± 0.43	12.51 ± 0.50	13.86 ± 0.68	13.87 ± 0.76	14.68 ± 0.33
	11.51–12.95; 7	11.70–13.60; 23	13.00–15.10; 9	12.90–15.10; 6	14.20–15.10; 4
MTW	24.68 ± 1.00	21.67 ± 1.57	25.58; 1	25.93 ± 1.18	25.60 ± 0.64
	23.66–26.38; 7	19.50–24.50; 21		24.30–28.30; 14	24.70–26.20; 4
GWN	3.07 ± 0.29	2.97 ± 0.29	2.96; 1	2.70 ± 0.21	4.30 ± 0.00
	2.70–3.51; 7	2.60–3.60; 23		2.37–2.94; 6	4.30–4.30; 3
BM1	19.54 ± 0.64	17.38 ± 0.75	20.20; 1	21.08 ± 0.66	21.43 ± 0.25
	19.20–20.27; 7	16.50–19.50; 21		20.30–22.30; 14	21.10–21.70; 3
LUTR	25.27 ± 0.51	24.65 ± 1.12	27.85 ± 1.25	27.25 ± 0.99	27.90 ± 1.02
	24.45–25.89; 7	21.40–26.10; 23	25.00–29; 13	25.70–29.02;14	26.70–29.10; 4
LBTR	22.32 ± 1.02	21.19 ± 0.78	24.30; 1	24.91 ± 0.70	24.85 ± 0.44
	21.31–24.16; 7	20.20–23.70; 21		23.40–25.70;14	24.20–25.30; 4

**Figure 3.**
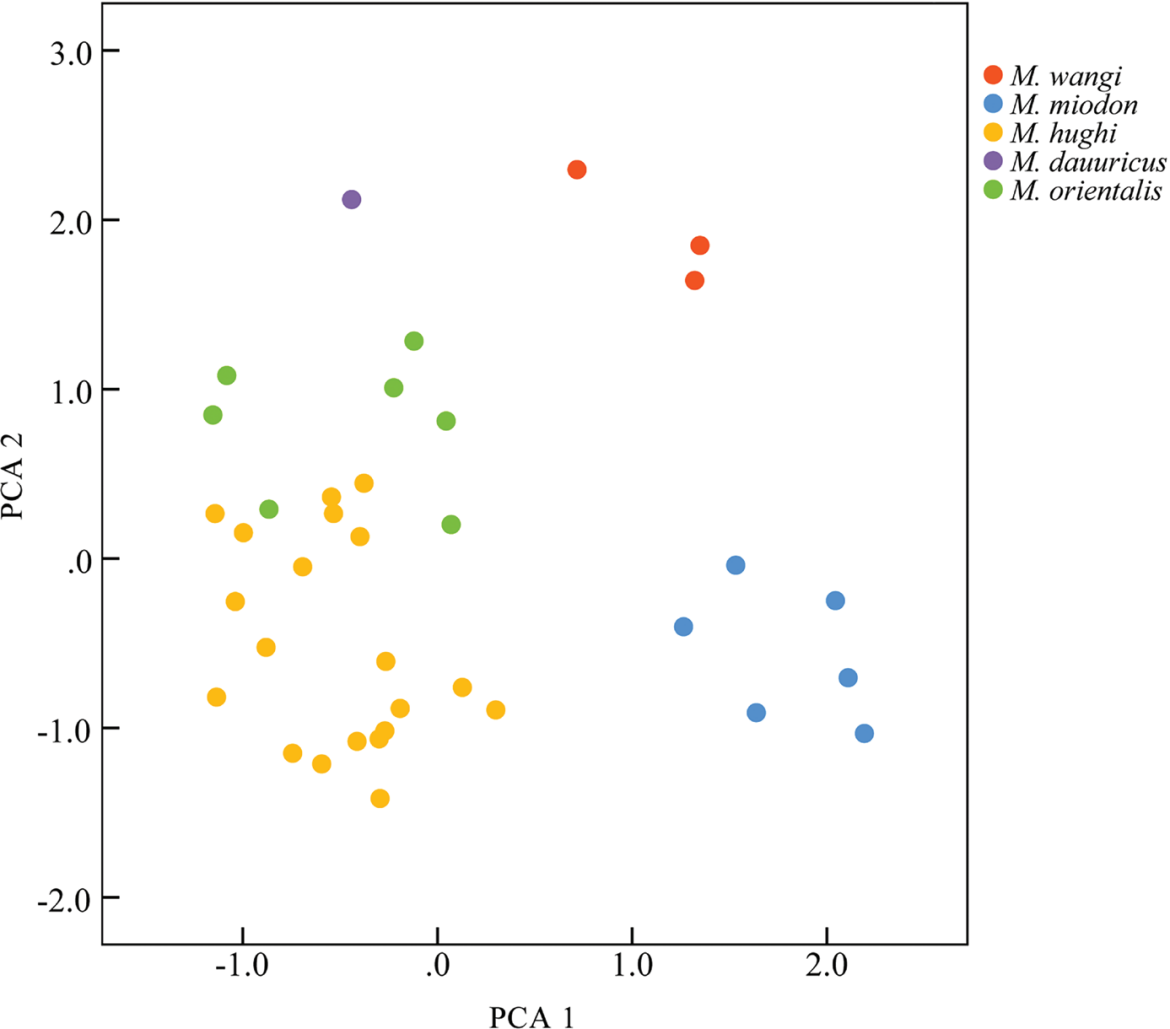
Plot of *Mesechinusorientalis* sp. nov. for PCA 1 and PCA 2.

The first two PCA axes had eigenvalues exceeding 1.0 (Table 3). The first principal component (PC1) accounted for 69.32% of the total variance and was positively correlated with all variables (loading > 0.69), reflecting a size effect. The second principal component (PC2) accounted for 10.01% of the variance and was strongly positively correlated with GWN, MTW, and BM1 (loading > 0.53). The PC1 vs PC2 scatter plot (Fig. 3) showed *M.orientalis* sp. nov. slightly overlapping with *M.hughi* but well separated from *M.wangi*, *M.dauuricus*, and *M.miodon*. Specimens of *M.orientalis* sp. nov. and *M.hughi* mainly occupy the negative regions of PC1, reflecting their smaller size compared to *M.wangi* and *M.miodon*. *Mesechinusorientalis* sp. nov. plotted on the positive regions of PC2, whereas most *M.hughi* specimens plotted on the negative regions, suggesting the new species has wider nasal, mastoid, and M^1^ than *M.hughi*.

**Table 3.** Factor loading eigenvalues and percentage of variance explained for PC1 and PC2 from principal component analysis.

Variables	Component	
	1	2
BL	0.964	−0.107
CBL	0.956	−0.143
GLS	0.944	−0.208
LUTR	0.922	−0.090
PL	0.910	0.165
LBTR	0.895	0.100
BM1	0.874	0.251
ZMB	0.832	0.020
IOB	0.791	−0.022
MTW	0.745	0.306
GWN	0.308	0.715
CH	0.591	−0.642
Eigenvalues	8.294	1.210
Variance explained (%)	69.116	10.081

### ﻿Morphological characteristic matrix

**Figure 4.**
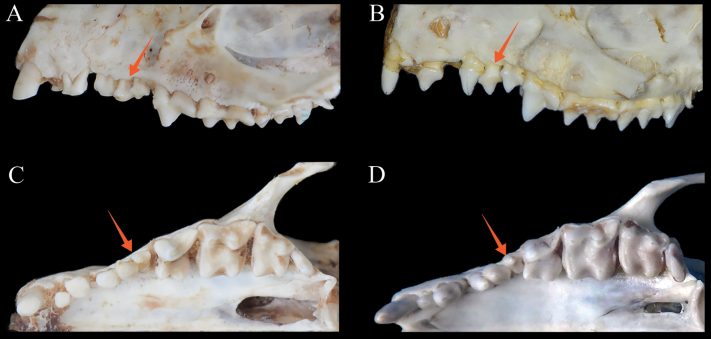
Ventral and lateral views of left upper toothrow of *M.orientalis* sp. nov. **A, C, B, D***M.hughi*. The arrows point at the root of P^2^ (**A, B**), and the protocone of P^3^ (**C, D**).

The morphological characteristics matrix is summarized in Suppl. material 3, and the specific characters represented by each number are interpreted in Suppl. material 4. Based on the matrix, *M.orientalis* sp. nov. differs from the most morphologically similar species, *M.hughi* in several characteristics: (1) the parietal is relatively higher than the frontals (frontals more elevated than parietals in *M.hughi*; character 32 in Suppl. material 3); (2) the posterior palatal spine is vestigial (vs spine is well developed in *M.hughi*; character 25); (3) suprameatal fossa is moderately developed (vs shallow in *M.hughi*; character 30); (4) P^2^ is two-rooted and not completely fused (Fig. 4) (the single root or two roots of P^2^ are well fused in *M.hughi*; character 73); (5) P^3^ is small because of a vestigial protocone (vs larger and with protocone well developed in *M.hughi*; character 82).

### ﻿Phylogenetic relationships

**Figure 5.**
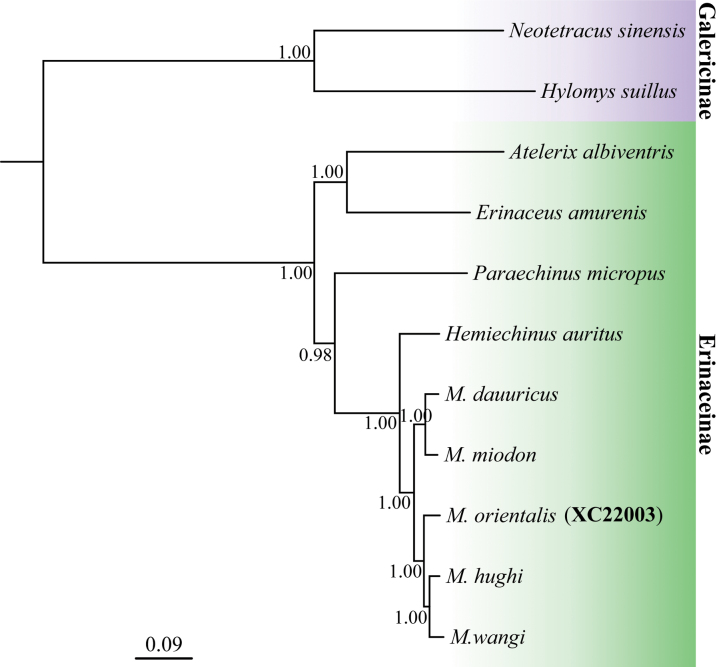
Mitochondrial gene tree of the genus *Mesechinus* and other genera of Erinaceinae and the outgroup. Branch lengths represent substitutions per site. Numbers above branches are posterior probability supporting the relationship.

The ML and BI trees showed identical topologies, and only the BI tree is shown (Fig. 5). Relationships among all *Mesechinus* species were strongly supported (PP = 1.00). *Mesechinusorientalis* sp. nov. is strongly supported as embedded within the *Mesechinus* clade (PP = 1.00). Among the *Mesechinus* species, the new species forms a sister relationship (PP = 1.00) to the *M.hughi* + *M.wangi* clade, whose sister relationship was also strongly supported (PP = 1.00).

### ﻿Divergence times

**Figure 6.**
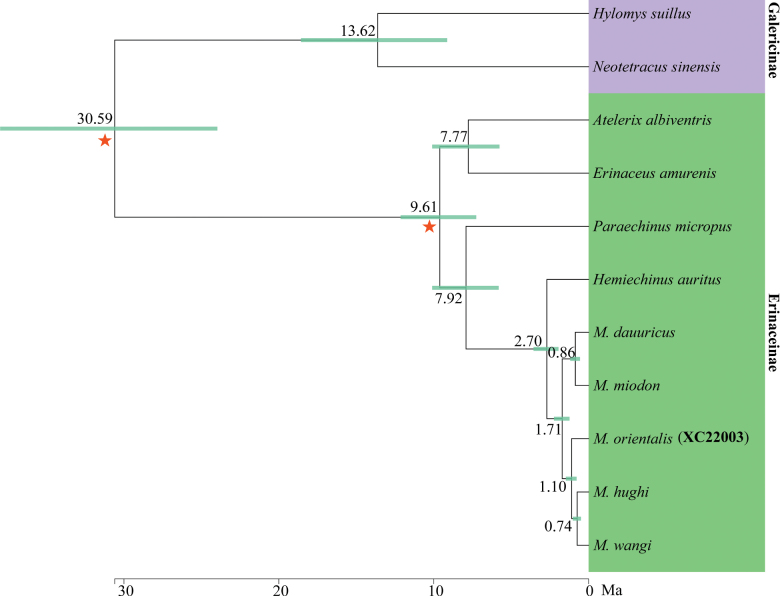
Divergence times estimated using BEAST based on mitogenome data. Branch lengths represent time. Numbers above branches refer to divergence time in millions of years. Asterisks indicate the location of correction points.

Divergence time estimates show that the most recent common ancestor of *Mesechinus* occurred in the early Pleistocene, ca 1.71 Ma (95% CI = 1.23–2.24 Ma) (Fig. 6). *Mesechinusorientalis* sp. nov. diverged from the *M.hughi* + *M.wangi* ancestor ca 1.10 Ma (95% CI = 0.78–1.47 Ma), with *M.hughi* and *M.wangi* diverging ca 0.74 Ma (95% CI = 0.50–1.02 Ma).

### ﻿Karyotypic characteristics of *Mesechinusorientalis* sp. nov.

**Figure 7.**
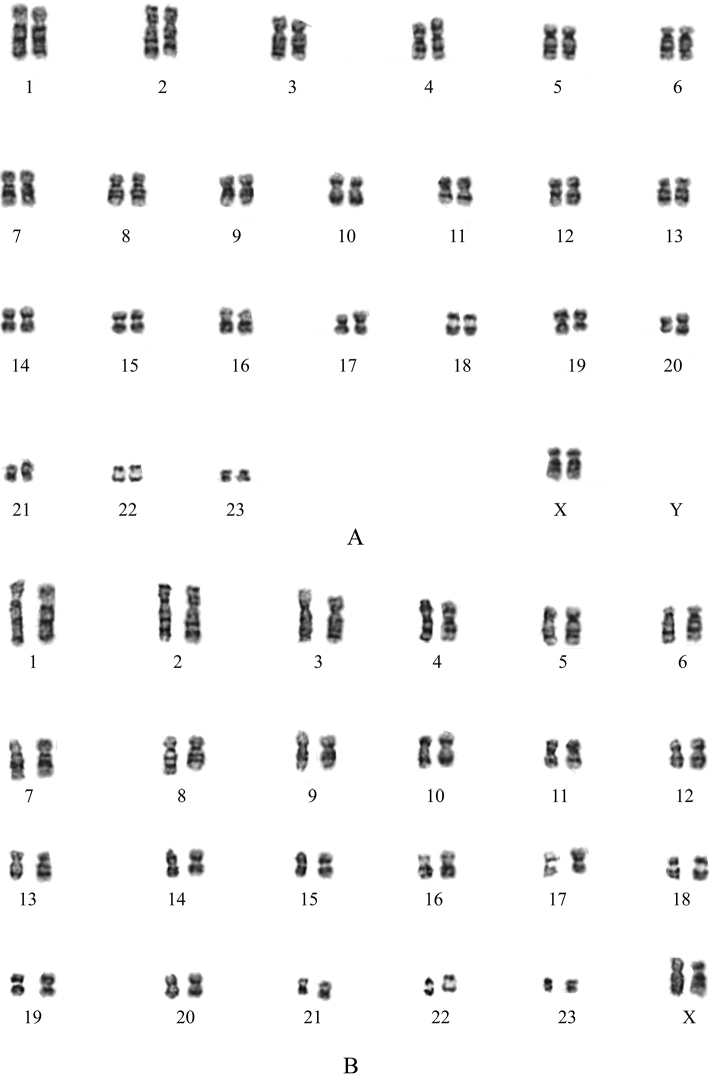
Comparison of karyotypes in two *Mesechinus* species **A***M.orientalis* sp. nov. (XC 2205003) **B***M.wangi*.

The comparison of the G-banding chromosomes of *M.orientalis* sp. nov. and *M.wangi* is shown in Fig. 7. Since the specimen was a female individual, its X chromosome was determined by comparing its karyotype with that of *M.wangi*. The two species’ autosomes are almost identical; the diploid number (2n) and autosomal fundamental number (FNa) are 48 and 92, respectively. The only difference was the X chromosome, where that of *M.wangi* was meta centric, while that of *M.orientalis* sp. nov. was submetacentric.

Based on the morphological, morphometric, and molecular evidence and the modern phylogenetic species concept (phylogenetic species concept based on both diagnosability and monophyly as operational criteria) (Mayden 1997; Gutiérrez and Garbino 2018), we recognize the *Mesechinus* population from Anhui as a new species and formally describe it below.

### ﻿Taxonomic account

#### ﻿ Mesechinus orientalis sp. nov.

Taxon classificationAnimaliaEulipotyphlaErinaceidae

D0FB49BA-C64C-5E96-9B69-C0640308DDCA

https://zoobank.org/BB3A29EC-F0A8-4DFD-A954-D5AC8E03B4B2

##### Suggested common name.

Eastern Forest Hedgehog, 华东林猬 (Huadong Linwei).

##### Type materials.

***Holotype***: XC 23001, an adult male collected from Xikou Town (30°34'42"N, 118°41'47"E), Xuancheng City, southern Anhui, China, Zifan Shi leg., May 2023. The dried skin, cleaned skull, and tissue samples are deposited in AHNU. ***Paratypes***: XC 18001, XC 2205001, XC 2205003, XC 2205005, XC 2205006, HZ 22001, six adult specimens collected from southeast Anhui and northwest Zhejiang, China, between 2018 and 2023. The specimens are deposited in AHNU.

##### Etymology.

The specific name *orientalis* is derived from the Latin *oriens*, “the east”, and suffix -*alis*, “pertaining to”, in reference to the new species’ eastern distribution in Anhui and Zhejiang provinces in eastern China.

##### Diagnosis.

**Figure 8.**
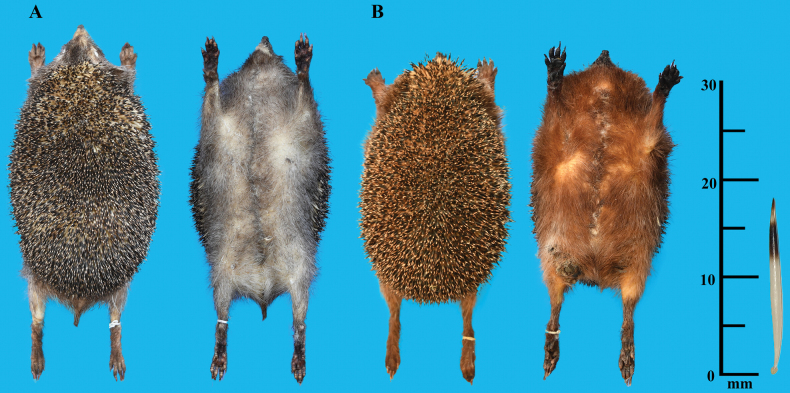
External morphs and spines of *Mesechinusorientalis* sp. nov. **A** male specimen (XC 2205001) **B** female specimen (XC 2205005).

**Figure 9.**
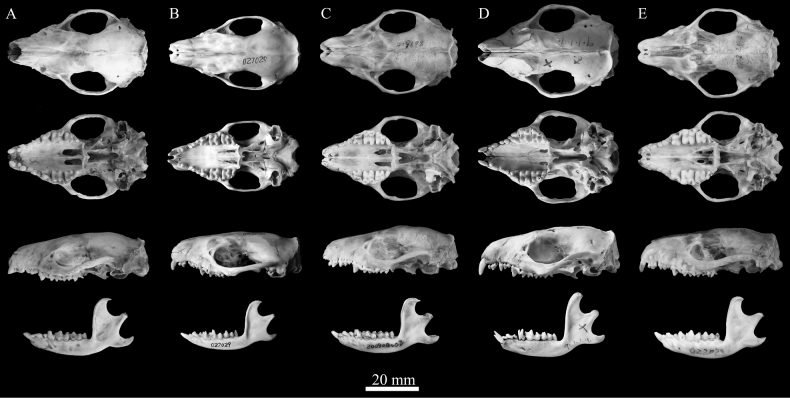
Dorsal ventral and lateral views of skull and mandible of *Mesechinus* species **A***M.orientalis* sp. nov. (holotype XC 23001) **B***M.hughi* (KIZ 027029) **C***M.dauuricus* (KIZ 027005) **D***M.miodon* (holotype BM 9.1.1.9) **E***M.wangi* (holotype KIZ 022028).

This is a small-bodied hedgehog (GLS = 49.95 ± 1.69 mm), similar to *M.hughi*, but smaller than other *Mesechinus* species. It has the shortest spines in the genus (18–20 mm); the spines have four-colour rings, similar to the spines of *M.dauuricus* and *M.hughi*, but different from those of *M.miodon* and *M.wangi* (Fig. 8). The parietal is higher than the frontals, which differs from that of *M.hughi* and *M.wangi* (Fig. 9). The P^2^ is two-rooted and not completely fused (Fig. 4). The protocone of P^3^ is vestigial, which differs from that of *M.hughi*, and smaller than P^2^, which distinguishes it from *M.dauuricus.* The dental formula of *M.orientalis* sp. nov. [I 3/2, C1/1, P 3/2, M 3/3 (×2) = 36], which distinguishes it from *M.wangi*.

##### Description.

This is a small-bodied *Mesechinus* species (HB = 188.83 mm; GLS = 49.95 mm) (Table 2). The ears are small and nearly the same length as the surrounding spines (Fig. 8). The nose is brown, with black whiskers on the snout; these whiskers shorten towards the nose. The spines are the shortest (18–20 mm) among *Mesechinus* species. There are four colour rings on the spine from the base to the tip: two-thirds of the length is white at the base, followed by a 3–4 mm black ring, a narrow light ring, and a black tip (Fig. 8). This species appears to be sexually dimorphic; among the specimens we collected, the pelage of males was generally grey, while that of most of the females (2 of 3 specimens) was reddish brown. However, this is a relatively small sample size, and further investigation is required to establish sexual dimorphism with more certainty.

The skull is heavy and with a shortened rostrum, and the lambdoidal crest is evident. The parietal is relatively higher than the frontal (Fig. 9). On the ventral side of the skull, a posterior palatal shelf and vestigial posterior palatal spine (<1 mm) extend slightly posteriorly. The suprameatal fossa is moderately developed and has the anterior and posterior rim nearly parallel, giving the fossa a more angular or U-shaped appearance. The zygomatic arches are significantly expanded, and the temporal fossa is large and subrounded. The middle palatine foramen is relatively larger than in other *Mesechinus* species. The coronoid process of the mandible is tall, rising upward from the posterior of the toothrow; the tips are slightly curved to the posterior, and the posterior surface is concave (Fig. 9). The mandibular condyle sits posteriorly below the coronoid process at a nearly 45° angle. The angular process is enlarged, thick, long, and triangulate.

As with other *Mesechinus* species, except *M.wangi* which has an additional M^4^, the dental formula of the new species is I 3/2, C1/1, P 3/2, M 3/3 (×2) = 36. The I^1^ is enlarged, I^2^ is much smaller than I^1^ and I^3^, and I^3^ has two roots. P^2^ also has two roots which are not completely fused. P^3^ is small (smaller than P^2^) and has a vestigial protocone. M^1^ is slightly larger than M^2^, and M^3^ is reduced.

##### Comparison.

The hedgehogs from China’s Anhui and Zhejiang provinces can be easily classified as *Mesechinus* based on the following morphological characteristics: the absence of pure white spines; relatively small ears, almost similar in length to the surrounding spines; no bare part on the forehead nor at the top of the forehead which divides the spines on the head into two halves; and a U-shaped suprameatal fossa.

Among the *Mesechinus* species, *M.orientalis* sp. nov. is morphologically most similar to *M.hughi.* However, the new species can be distinguished by many characters. *Mesechinusorientalis* sp. nov. has the shortest spines in the genus (18–20 mm), shorter than those in *M.hughi* (22–24 mm). The parietal is relatively higher than the frontals in the new species, whereas the frontals are relatively higher than parietals in *M.hughi*. P^2^ has two roots which are not completely fused in *M.orientalis* sp. nov., while in *M.hughi* P^2^ the two roots are well fused. The P^3^ protocone is vestigial in the new species but well developed in *M.hughi*. The posterior palatal spine is vestigial, and the suprameatal fossa is moderately developed in the new species, which differs from the well-developed posterior palatal spine and shallow suprameatal fossa in *M.hughi.* In addition, the MTW and BM^1^ of the new species are significantly greater than those of *M.hughi* (*P* < 0.01).

*Mesechinusorientalis* sp. nov. (HB = 188.83 mm ± 8.13; GLS = 49.95 mm ± 1.69) is distinguishable from *M.dauuricus* (HB = 373.91 mm ± 21.35; GLS = 55.18 mm ± 3.07), *M.miodon* (HB = 205 mm ± 23.53; GLS = 54.10 mm ± 2.10), and *M.wangi* (HB = 208.75 mm ± 21.90; GLS = 54.75 mm ± 0.70) by its smaller size. The spines of the new species are much shorter (18–20 mm) than those of *M.dauuricus* (21–24 mm), *M.miodon* (~26 mm), and *M.wangi* (21–24 mm). The spines of *M.orientalis* sp. nov. have four-colour rings similar to those of *M.dauuricus* and *M.hughi*, but they differ from *M.miodon* and *M.wangi*. P^3^ of the new species is much smaller than P^2^, which differs from *M.dauuricus*, in which P^3^ is of equal size to P^2^. The parietal is relatively higher than the frontals in *M.orientalis* sp. nov., which differs from *M.wangi*. Additionally, the presence of M ^4^ in *M.wangi* is unique in the genus, which easily distinguishes it from other species.

##### Distribution and habitat.

*Mesechinusorientalis* sp. nov. is currently known from southern Anhui (Xuancheng and Huangshan) and northwestern Zhejiang (Anji, Changxing, Deqing, Yuhang, Linan, Chunan), both in eastern China. Most specimens were collected in scrubland and subtropical broad-leaf evergreen forests at elevations from 30 to 700 m a.s.l.

## ﻿Discussion

For a long time, the genus *Mesechinus* was thought to be restricted to northern China and adjacent Mongolia and Russia (Wilson and Reeder 2005) until Ai (2007) reported a small population of *Mesechinus* from Mount Gaoligong in Yunnan Province, southwestern China. This Mount Gaoligong population was subsequently described as a new species, *M.wangi* (Ai et al. 2018). In this study, we recognized a population of *Mesechinus* from eastern China as another isolated species, *M.orientalis* sp. nov. Morphologically, the new species is most similar to *M.hughi*, but it is distinguishable from all recognized *Mesechinus* species in having the shortest spines, an incompletely fused two-rooted P^2^, and a smaller, vestigial P^3^. The new species is geographically isolated from its congeners by at least 1000 km, and it is currently the southeasternmost species of *Mesechinus* (Fig. 1). Except for *M.orientalis* sp. nov., only one species of hedgehog, *Erinaceusamurensis* Schrenk, 1859, occurs in eastern China. While these species are sympatric, at least in Xuancheng, Anhui Province, *M.orientalis* sp. nov. can easily be distinguished from *E.amurensis* by the absence of pure-white spines, and no bare part on the forehead nor at the top of the forehead which divides the spines on the head into two halves (Fig. 8).

The discovery of a new species of *Mesechinus* in eastern China has greatly expanded the known range of the genus and is vital in understanding the macroevolution of the genus. The oldest fossils of *Mesechinus* are from the Early Pleistocene near Taijiaping Village in Nangaoya Township, Tianzhen, Shanxi Province (Bai et al. 2022). Our molecular results reveal that the divergences among *M.orientalis*, *M.wangi*, and *M.hughi* occurred in the Middle Pleistocene 0.74–1.10 Ma (Fig. 6). Increased cooling and aridification during the middle Pleistocene (known as the middle Pleistocene transition at ca 1.2–0.5 Ma) appear to have been critically important in the split of the three species, which may also have facilitated the migration of the ancestors of *M.wangi* and *M.orientalis* sp. nov. to southwestern and southeastern China, respectively. The north–south trending Dabie Mountains, which are located between the Qinling Mountains and southern Anhui, may have provided a migration route for the ancestor of *M.orientalis* sp. nov. to reach southern Anhui. The mountainous area of southern Anhui and northwestern Zhejiang Province also likely acted as glacial refugia in the Pleistocene for the new species.

## Supplementary Material

XML Treatment for Mesechinus orientalis

## ﻿Additional information

### Author ORCIDs

Zifan Shi https://orcid.org/0009-0008-5165-6604

Kai He https://orcid.org/0000-0002-6234-2589

Jiajun Zhou https://orcid.org/0000-0003-1038-1540

Kenneth O. Onditi https://orcid.org/0000-0003-4034-6818

Xuelong Jiang https://orcid.org/0000-0003-2052-2490

Zhongzheng Chen https://orcid.org/0000-0003-3821-0145

### Data availability

All of the data that support the findings of this study are available in the main text or Supplementary Information.

## Supplementary materials

Supplementary material 1 Collection information of *Mesechinusorientalis* sp. nov. Data type xlsx https://binary.pensoft.net/file/943945This dataset is made available under the Open Database License (http://opendatacommons.org/licenses/odbl/1.0/). The Open Database License (ODbL) is a license agreement intended to allow users to freely share, modify, and use this Dataset while maintaining this same freedom for others, provided that the original source and author(s) are credited.Zifan Shi, Hongfeng Yao, Kai He, Weipeng Bai, Jiajun Zhou, Jingyi Fan, Weiting Su, Wenhui Nie, Shuzhen Yang, Kenneth O. Onditi, Xuelong Jiang, Zhongzheng Chen

Supplementary material 2 Partitioning schemes used in mitogenome RAxML analyses Data type xlsx https://binary.pensoft.net/file/943946This dataset is made available under the Open Database License (http://opendatacommons.org/licenses/odbl/1.0/). The Open Database License (ODbL) is a license agreement intended to allow users to freely share, modify, and use this Dataset while maintaining this same freedom for others, provided that the original source and author(s) are credited.Author: Zifan Shi

Supplementary material 3 Morphological characteristic matrix Data type xlsx Explanation note Matrix of morphological characters of erinaceid species. The specific characters represented by each number are interpreted in Suppl. material 4. https://binary.pensoft.net/file/943947This dataset is made available under the Open Database License (http://opendatacommons.org/licenses/odbl/1.0/). The Open Database License (ODbL) is a license agreement intended to allow users to freely share, modify, and use this Dataset while maintaining this same freedom for others, provided that the original source and author(s) are credited.Zifan Shi, Hongfeng Yao, Kai He, Weipeng Bai, Jiajun Zhou, Jingyi Fan, Weiting Su, Wenhui Nie, Shuzhen Yang, Kenneth O. Onditi, Xuelong Jiang, Zhongzheng Chen

Supplementary material 4 Morphological transformation series Data type doc https://binary.pensoft.net/file/943948This dataset is made available under the Open Database License (http://opendatacommons.org/licenses/odbl/1.0/). The Open Database License (ODbL) is a license agreement intended to allow users to freely share, modify, and use this Dataset while maintaining this same freedom for others, provided that the original source and author(s) are credited.Zifan Shi, Hongfeng Yao, Kai He, Weipeng Bai, Jiajun Zhou, Jingyi Fan, Weiting Su, Wenhui Nie, Shuzhen Yang, Kenneth O. Onditi, Xuelong Jiang, Zhongzheng Chen

## References

[B1] Ai HS (2007) A short introduction of the cover photo for the current issue. Zoological Research 28(6): 633. https://www.zoores.ac.cn/en/article/id/39

[B2] Ai HS He K Chen ZZ Li JQ Wan T Li Q Nie WH Wang JH Su WT Jiang XL (2018) Taxonomic revision of the genus *Mesechinus* (Mammalia: Erinaceidae) with description of a new species. Zoological Research 39: 335–347. 10.24272/j.issn.2095-8137.2018.034 29695683 PMC6102679

[B3] Andreychev A Kuznetsov V (2020) Checklist of rodents and insectivores of the Mordovia, Russia. ZooKeys 1004: 129–139. 10.3897/zookeys.1004.57359 33384568 PMC7758312

[B4] Bai W Dong W Liu W Zhang L Li L Li Q (2022) Pleistocene Hedgehog *Mesechinus* (Eulipotyphla, Mammalia) in China. Journal of Mammalian Evolution 29(4): 797–814. 10.1007/s10914-022-09612-w

[B5] Bannikova AA Matveev VA Kramerov DA (2002) Using inter-SINE–PCR to study mammalian phylogeny. Russian Journal of Genetics 38(6): 714–724. 10.1023/A:1016056304555 12138785

[B6] Bernt M Donath A Jühling F Externbrink F Florentz C Fritzsch G Pütz J Middendorf M Stadler PF (2013) MITOS: Improved de novo metazoan mitochondrial genome annotation. Molecular Phylogenetics and Evolution 69(2): 313–319. 10.1016/j.ympev.2012.08.023 22982435

[B7] Bouckaert R Heled J Kühnert D Vaughan T Wu CH Xie D Suchard MA Rambaut A Drummond AJ (2014) BEAST 2: A software platform for Bayesian evolutionary analysis. PLoS Computational Biology 10(4): e1003537. 10.1371/journal.pcbi.1003537 PMC3985171 24722319

[B8] Chen ZZ Tang XF Tang HY Zhao HT Miao QL Shi ZF Wu HL (2020) First record of genus *Mesechinus* (Mammalia: Erinaceidae) in Anhui Province, China— *Mesechinushughi*. Acta Theriologica Sinica 40: 96–99. http://www.mammal.cn/CN/10.16829/j.slxb.150318

[B9] Corbet GB (1988) The family Erinaceidae: A synthesis of its taxonomy, phylogeny, ecology and zoogeography. Mammal Review 18(3): 117–172. 10.1111/j.1365-2907.1988.tb00082.x

[B10] de Sena Brandine G Smith AD (2021) Falco: High-speed FastQC emulation for quality control of sequencing data. F1000 Research 8: 1874. 10.12688/f1000research.21142.2 PMC7845152 33552473

[B11] Dierckxsens N Mardulyn P Smits G (2017) NOVOPlasty: De novo assembly of organelle genomes from whole genome data. Nucleic Acids Research 45: e18. 10.1093/nar/gkw955 PMC5389512 28204566

[B12] Frost DR Wozencraft WC Hoffmann RS (1991) Phylogenetic relationships of hedgehogs and gymnures (Mammalia, Insectivora, Erinaceidae). Smithsonian Contributions to Zoology 518(518): 1–69. 10.5479/si.00810282.518

[B13] Gould GC (1995) Hedgehog phylogeny (Mammalia, Erinaceidae)—The reciprocal illumination of the quick and the dead. American Museum Novitates 3131: 1–45. http://hdl.handle.net/2246/3665

[B14] Gutiérrez EE Garbino GS (2018) Species delimitation based on diagnosis and monophyly, and its importance for advancing mammalian taxonomy. Zoological Research 39: 301–308. 10.24272/j.issn.2095-8137.2018.037 29551763 PMC6102684

[B15] He K Eastman TG Czolacz H Li S Shinohara A Kawada S Springer MS Berenbrink M Campbell KL (2021) Myoglobin primary structure reveals multiple convergent transitions to semi-aquatic life in the world’s smallest mammalian divers. eLife 10: e66797. 10.7554/eLife.66797 PMC8205494 33949308

[B16] Kearse M Moir R Wilson A Stones-Havas S Cheung M Sturrock S Buxton S Cooper A Markowitz S Duran C Thierer T Ashton B Meintjes P Drummond A (2012) Geneious Basic: An integrated and extendable desktop software platform for the organization and analysis of sequence data. Bioinformatics 28(12): 1647–1649. 10.1093/bioinformatics/bts199 22543367 PMC3371832

[B17] Korablev VP Kirilyuk V Golovushkin MI (1996) Study of the karyotype of daurian hedgehog *Mesechinusdauuricus* (mammalia, erinaceidae) from its terra typica. Zoologicheskii Zhurnal [Зоологический журнал] 75: 563–564.

[B18] Kryštufek B Motokawa M (2018) Talpidae (Moles, desmans, star-nosed moles and shrew moles). In: Wilson D Mittermeier R (Eds) Handbook of the Mammals of the World.Vol. 8: Insectivores, Sloths, Colugos. Lynx Editions, Barcelona, 552–620.

[B19] Lanfear R Frandsen PB Wright AM Senfeld T Calcott B (2017) PartitionFinder 2: New methods for selecting partitioned models of evolution for molecular and morphological phylogenetic analyses. Molecular Biology and Evolution 34: 772–773. 10.1093/molbev/msw260 28013191

[B20] Mayden R (1997) A hierarchy of species concepts: The denouement in the saga of the species problem. Systematics Association Special 54: 381–423.

[B21] Meng G Li Y Yang C Liu S (2019) MitoZ: A toolkit for animal mitochondrial genome assembly, annotation and visualization. Nucleic Acids Research 47(11): e63–e63. 10.1093/nar/gkz173 PMC6582343 30864657

[B22] Meredith RW Janečka JE Gatesy J Ryder OA Fisher CA Teeling EC Goodbla A Eizirik E Simão TL Stadler T Rabosky DL Honeycutt RL Flynn JJ Ingram CM Steiner C Williams TL Robinson TJ Burk-Herrick A Westerman M Ayoub NA Springer MS Murphy WJ (2011) Impacts of the Cretaceous Terrestrial Revolution and KPg extinction on mammal diversification. Science 334(6055): 521–524. 10.1126/science.1211028 21940861

[B23] Miller MA Schwartz T Pickett BE He S Klem EB Scheuermann RH Passarotti M Kaufman S O’Leary MA (2015) A RESTful API for access to phylogenetic tools via the CIPRES science gateway. Evolutionary Bioinformatics 2015: 11. [EBO S21501] 10.4137/EBO.S21501 PMC4362911 25861210

[B24] Müller S (1840) Over de zoogdieren van den Indischen Archipel. In: Temminck CJ (Ed.) Verhandelingen over de natuurlijke geschiedenis der Nederlandsche overzeesche bezittingen, de Leden der natuurkundige commissie in Indiö en andere Schrijvers.Vol. 3, Zoology. J. Luchtmans en C. C. van der Hoek, Leiden, 9–57.

[B25] Pan QH Wang YX Yan K (2007) A Field Guide to the Mammals of China. Chinese Forestry Publishing House, Beijing, 420 pp.

[B26] Pavlinov I Rossolimo OL (1987) Geographic variation and intraspecific taxonomy of sable (*Marteszibellina* L.) in the USSR. Mammals. Studying the Soviet Union Fauna 18: 241–256.

[B27] Rambaut A (2017) FigTree-version 1.4. 3, a graphical viewer of phylogenetic trees. http://tree.bio.ed.ac.uk/software/Figtree

[B28] Rambaut A Drummond AJ Xie D Baele G Suchard MA (2018) Posterior summarization in Bayesian phylogenetics using Tracer 1.7. Systematic Biology 67(5): 901–904. 10.1093/sysbio/syy032 29718447 PMC6101584

[B29] Schrenk LV (1859) Reisen und Forschungen im Amur-Lande in den Jahren 1854–1856. Vol. Bd.1, Commissionäre der K. Akademie der Wissenschaften, St. Petersburg, 212 pp. 10.5962/bhl.title.15761

[B30] Sikes RS (2016) Guidelines of the American Society of Mammalogists for the use of wild mammals in research and education. Journal of Mammalogy 97(3): 663–688. 10.1093/jmammal/gyw078 29692469 PMC5909806

[B31] Sundevall CJ (1842) Ofversigt af slagtet *Erinaceus*. Kunglica Svenska Vetenskapsa-Akademiens. Handlingar 1841: 215–239.

[B32] Tamura K Stecher G Kumar S (2021) MEGA 11: Molecular Evolutionary Genetics Analysis version 11. Molecular Biology and Evolution 38(7): 3022–3027. 10.1093/molbev/msab120 33892491 PMC8233496

[B33] Thomas O (1908) The Duke of Bedford’s Zoological Exploration in Eastern Asia.—XI. On mammals from the provinces of Shan‐si and Shen‐si, northern China. Proceedings of the Zoological Society of London 78(4): 963–983. 10.1111/j.1469-7998.1908.00963.x

[B34] Trouessart EL (1909) *Neotetracussinensis*, a new insectivore of the family Erinaceidae. The Annals and Magazine of Natural History (Series 8) 4: 389–391. 10.1080/00222930908692683

[B35] Wilson DE Mittermeier RA (2018) Handbook of the Mammals of the World. Vol. 8: Insectivores, Sloths and Colugos. Lynx Edicions, Barcelona, 326–327.

[B36] Wilson DE Reeder DM (2005) Mammal Species of the World: a Taxonomic and Geographic Reference. Vol. 1. JHU Press, Baltimore, 2, 142 pp.

[B37] Xie J Chen Y Cai G Cai R Hu Z Wang H (2023) Tree visualization by one table (tvBOT): A web application for visualizing, modifying and annotating phylogenetic trees. Nucleic Acids Research 51(W1): 587–592. 10.1093/nar/gkad359 PMC10320113 37144476

[B38] Zhang D Gao F Jakovlić I Zou H Zhang J Li WX Wang GT (2020) PhyloSuite: An integrated and scalable desktop platform for streamlined molecular sequence data management and evolutionary phylogenetics studies. Molecular Ecology Resources 20(1): 348–355. 10.1111/1755-0998.13096 31599058

