# Supplementary material for: ﻿A new species of forest hedgehog (Mesechinus, Erinaceidae, Eulipotyphla, Mammalia) from eastern China
Source: Zookeys. 2023 Nov 28;1185:143–61. doi: 10.3897/zookeys.1185.111615 (PMC10698749; doi:10.3897/zookeys.1185.111615)
Supplement: Supplementary material 4 — Morphological transformation series [file zookeys-1185-143_article-111615__-s004.doc]

**Table S4 Morphological transformation series**

Below is a list of transformation series (TS) reviewed in this analysis. All characters, unless otherwise indicated, were taken from Frost et al. [1], and Gould [2] and Gould [3], and denoted as (#), (†), and (‡), respectively. Those left unmarked, indicate that the numbering was congruent across data sets. The characters are numbered in a manner that essentially follows previous character lists. So character 1, is the first character in all three previous analyses. Where characters deviate from this have now noted alongside our character number. Finally, several new transformation series were added along with a few new character states, they are indicated as (new). In some cases, a character state was added to the transformation series to accommodate a fossil taxon, which is also noted.

All transformation series are ordered unless otherwise specified. For in depth discussions of each transformation series and the rationale for ordering them, please refer to the original sources. None of the dental transformation series, however, were ordered because Gould [3] suggested that dental characters were quite plastic across all of the living taxa. This is not to say that there is no phylogenetic signal, only that when considered alone (in absence of cranial characters) in a phylogenetic analysis, they resulted in considerable noise.

In several of the analyses we looked at the effects of weighting cranial, postcranial and pelage characters over dental characters because of the noise that dental characters contribute to the analysis [3]. Weighted characters in this analyses included 1-61, and 121-135. Characters excluded in phylogenetic analyses are 65, 67-69, 75-80, 101-107, and 115-120.

**Cranial Characters:**

1. Nasals, posterior extension: (0) anterior the antorbital rim; (1) medial or posterior to antorbital rim.
2. Rostrum length: (0) long; (1) short.
3. Rostrum width: (0) wide, anterior incisors not closely adjacent; (1) narrow, anterior incisors not closely adjacent.
4. Palatine foramina, size: (0) small; (1) anterior foramina elongated posteriorly; (2) anterior and medial foramina coalesced.
5. Anterior palatine foramen, location: (0) at the maxilla/palatine suture; (1) anterior to the maxilla/palatine suture.
6. Position of anterior opening of the infraorbital canal: (0) dorsal or posterodorsal to P^4^-M^1^; (1) dorsal to P^3^-P^4^.
7. Antorbital fossa: (0) present; (1) absent.
8. Antorbital flange and lacrimal canal: (0) lacrimal canal visable from lateral view; (1) lacrimal canal obstructed from lateral view by antorbital flange.
9. (†9) Location of antoribtal (prelacrimal) flange: (0) anteroventral to lacrimal canal; (1) flange arcs round the anterior rim of the lacrimal canal; (2) anterodorsal to lacrimal canal; (3) absent.
10. (#9) Postventral process of the zygoma (maxilla): (0) absent; (1) present, small; (2) large.
11. (#10) Jugal size: (0) large, reaches lacrimal; (1) small; does not reach lacrimal; (2) vestigial, confined to lacrimal rim of zygoma; (3) absent. Unordered.
12. (#11) Pterygoid/alispenoid and eptierygoid processes: (0) epiterygoid processes absent, alisphenoid not inflated; (1) epiterygoid processes present, alisphenoid not inflated; (2) epiterygoid processes present, alisphenoid inflated. Unordered. See also Gould [2] TS†12 for an expanded discussion.
13. (#12) Lacrimal/maxilla suture: (0) unfused; (1) fused.
14. **(#13)** **Supraorbital process, a frontal process on the parietal/frontal suture: (0) absent, or poorly defined; (1) present, sharp.**
15. **(#14) Supraorbital foramen (frontal): (0) present; (1) absent.**
16. **Anterior process of the parietal: (0) absent, or very weak; (1) extends anteriorly** **along the supraorbital rim to form the base of the supraorbital process.**
17. Anterior process of the alisphenoid: (0) absent; (1) present.
18. Anterior opening of the inferior stapedial foramen (alisphenoid): (0) inferior ramus of the stapedial artery emerges from the bulla via a groove; (1) artery emerges from a foramen in the alisphenoid.
19. Cranio-orbital foramen (frontal): (0) closely associated or joined with the ethmoid foramen; (1) craniorbital and ethmoid foramina are widely separated. (Note: see Gould [2] for more comments about this TS.)
20. Suboptic foramen (orbitsphenoid): (0) absent; (1) present. (Note: see Gould [2] for more comments about this TS.)
21. (†21) Suboptic foramen (orbitosphenoid), relative position: (0) anterior to sphenorbital fissure; (1) located in the medial wall of the sphenorbital; (2) located in the medial wall of the sphenorbital fissure but hidden within from lateral view by the alisphenoid anterior process. Unordered.
22. (†22) Alisphenoid canal: (0) present; (1) absent, or very weakly developed (only exhibited on one side of the cranium).
23. (†23) Alisphenoid/orbitalsphenoid bridge: (0) absent or incomplete; (1) present, strongly developed. (Note: see Gould, [4] for more comments about this TS.)
24. (#21) Sphenopalatine foramen (palatine): (0) anterdorsal, or slightly posterodorsal to the palatine transverse torus; (1) decidedly posterodorsal to the torus.
25. **(#22) Palatal shelf and spine: (0) well-developed spine on posterior palatal shelf; (1) posterior spine absent or vestigial.**
26. (#23) Palatine, lateral fossa, anterodorsal to the palatine transverse torus: (0) absent; (1) present.
27. (#24) Zygomatic process of the squamosal: (0) not elevated posteriorly: (1) elevated posteriorly.
28. (#25) Postglenoid foramen (squamosal): (0) separated from the glenoid fossa by the entoglenoid process; (1) not separated.
29. (#26) Suprameatal fossa composition (squamosal): (0) mastoid and squamosal contributions equal; (1) squamosal and mastoid contributions subequal to predominately mastoid; (2) predominately squamosal contribution. State (2) was added by Gould [2] to accommodate *Neurogymnurus*, a fossil taxon from the Miocene. (Note: see Gould [2], TS†29 for more comments about this TS.)
30. (#27) Suprameatal fossa depth: (0) absent; (1) shallow; (2) moderately developed; (3) deep; (4) very deep.
31. (#28) Suprameatal fossa shape: (0) normal, anterior and posterior borders are widely separated; (1) compressed. (Note: see Gould [4], TS†31 for more comments about this TS.)
32. (#29) Relative height of skull: (0) parietals relatively higher than frontals; (1) frontals more elevated than parietals.
33. (#30) Nasophyngeal fossa (basisphenoid: (0) absent; (1) present.
34. (#31) Inflation of the basisphenoid: (0) absent; (1) present.
35. (†35) Temporal/sagital crest extended to frontal bones: (0) present, very strong; (1) absent or weak.
36. (new) Glenoid fossa, shape: (0) extension of fossa is anteroposteriorly directed, large articulation surface; (1) anteriorposteriorly extension reduced, lateral-medial extension increased; (2) no anteriorposterior extension, only lateral-medial extension. Unordered. Added by Gould [4] to accommodate several fossil taxa.
37. (new) Frontal crest: (0) absent; (1) merges with the antorbital flange; (2) becomes its own process, which is characterized by a ‘brow’ look above the orbit. Unordered. Added by Gould [4] to accommodate several fossil taxa.
38. (†36) Extreme flattening and lateral extension of the posterior region of the zygoma: (0) absent; (1) present. This TS was added by Gould [2] to accommodates *Proterix*, a Oligocene taxon.
39. (†37) Exostosis (rugosity) on the squamosal and parietal: (0) absent; (1) present, visibly noticeable; (2) present, well pronounced. State (2) was added by Gould [2] to accommodate *Neurogymnurus* and *Gymnurechinus*.
40. (#32) Ectotympanic: (0) slender, ring shaped, loosely attached with a small anterior process; (1) broader, sometimes engulfs anterior process, firmly attached.
41. (#33) Petrosal: (0) promontorium predominately confined to bullar (= tympanic) roof, squamosal does not participate in bullar roof; (1) promontorium forms posteromedial wall, squamosal is a major component of bullar roof; (2) promontorium forms posteromedial wall, mastoid and alispenoid are major contributors to bullar roof. Unordered. State (2) was added by Gould [2] to accommodate *Neurogymnurus*.
42. (#34) Inflation of the mastoid region between the exoccipital and squamosal: (0) absent; (1) present.
43. (#33/†41) Mastoid portion of suprameatal process: (0) not inflated; (1) inflated.
44. (#36) Ventral process of the petrosal: (0) not inflated; (1) inflated.
45. (#37) Basioccipital/petrosal suture: (0) narrow slit exposing portion of the interior petrosal sinus; (1) basioccipital/petrosal suture closed with a well defined posterior lacerate foramen.
46. (#38) Superior stapedial foramen (petrosal) location: (0) posterior to squamosal/alisphenoid suture and posterior to postglenoid foramen; (1) on squamosal/alisphenoid suture and close to the postglenoid foramen; (2) posterior to the alisphenoid/mastoid suture. Unordered. State (2) was added by Gould [2] to accommodate *Neurogymnurus*.
47. (#39) Epitympanic recess, lateral wall: (0) formed partially by squamosal; (1) formed entirely by mastoid; (2) formed entirely by squamosal. Unordered. State (2) was added by Gould [4] to accommodate the fossil taxa *Neurogymnurus*, *Deinogalerix*, and *Amphechinus edwardsi*.
48. (†46) Rostral tympanic process (RTP): (0) absent; (1) present. This TS is uninformative
49. (†47) Size of rostral tympanic process: (0) small; (1) large. This TS is uninformative
50. (†48) Position of rostral tympanic process: (0) restricted to medial aspect of promontorium; (1) situated on promontorium, but medially inflated; (2) situated medial to promontorium from which it spreads. Unordered. This TS is uninformative
51. (†49) Articular surface of the rostral tympanic process: (0) absent; (1) present.
52. (†50) Contribution of rostral tympanic process to the external auditory meatus: (0) absent; (1) present. This TS is uninformative.
53. (†51) Caudal tympanic process of petrosal: (0) absent or insignificant; (1) present; small, incompletely encloses fossula of fenestral cochleae; (2) present; extensive, delimits or forms apertures for the facial never (VII) and interal carotid artery. Unordered. This TS is uninformative.
54. (†52) Tympanic process of basiphenoid: (0) absent; (1) present, large; (2) present, large, coalesces with the alisphenoid with tympanic wing and forms the anteroinferior floor of the meatus. Unordered.
55. (†53) Tympanic process of the alisphenoid: (0) small, does not completely form tubal foramen in combination with the basisphenoid, little or not pneumatization; (1) large, completely forms tubal foramen combination with the basisphenoid, strongly pneumatized. This TS is uninformative.
56. (†54) Ectotympanic: (0) crura expanded relative to ontogentically early condition, ectotympanic phaneric (=hidden) or semiphaneric; (1) crura not expanded relative to ontogentically early condition, ectotympanic aphaneric, or highly semiphaneric. This TS is uninformative.
57. (†55) Arterial canals, intratympanic: (0) none or they only enclose stem of unreduced internal carotid at its entrance into tympanic cavity; (1) canals enclose stem of unreduced internal carotid and its major intratympanic divisions. This TS is uninformative.
58. (#40) Paroccipital process: (0) small; (1) robust.
59. (#41) Exoccipital: (0) not expanded; (1) expanded.
60. (#42) Occipital condyle: (0) condyle emarginated, looks loved; (1) no emargination.
61. (#44) Coronoid process: (0) narrow, pointed; (1) broad, rounded; (2) broad, rounded, vertical extension abbreviated. State (2) was added by Gould (Dissertation, 2007) to accommodate the fossil *Deinogalerix*. Unordered.

**Dental Characters:**

1. (‡4&5) I^1^: (0) present, enlarged; (1) present, small; (2) absent. Unordered.
2. (#45&46) I_1_: (0) present, enlarged; (1) present, small; (2) absent. Unordered.
3. (#48) I^2^, relative size: (0) greatly enlarged; (1) nearly equal to other incisors; (2) smaller than other incisors. Unordered. (Note, See Gould [2], TS†60 for an expanded discussion on this TS.)
4. *(‡149) I_2_, anterior midline crest: (0) ends posterior to protoconid; (1) ends anterior to protoconid; (2) absent. Unordered.*
5. (#48) I^2^; (0) greater than I^3^; (1) less than or equal to I^3^; (2) absent. Unordered.
6. *(#49) I^3^, number of roots: (0) one; (1) two, separate; (2) two, fused; (3) I^3^ absent. Unordered.*
7. *(‡151) I_3_, size: (0) smaller than other incisors; (1) approximately equal to other incisors; (2) larger than other incisors. Unordered.*
8. *(#52) Lower canine, relative size: (0) approximately equal to, or small than P_1_, P_2_, and P_3_: (1) significantly larger than P_1_, P_2_, and P_3_. Unordered.*
9. (#54) P^1^: (0) present; (1) absent.
10. (#55) P_1_: (0) present; (1) absent.
11. (†70) P_2_: (0) moderate size, two roots; (1) small, peg-like, procumbent, one root; (2) absent. Unordered. This TS is uninformative.
12. (#55) P^2^; (0) present, two roots; (1) present; one root or two roots well fused; (2) absent. Unordered. (Note, See Gould [2] TS†71 for an expanded discussion on this TS.)
13. (#57) P_3_: (0) larger in size than P_2_, two roots; (1) smaller in size than P_2_, one root; (2) absent. Unordered. Unordered*.*
14. *(‡175) P_3_, cusps: (0) one; (1) two; (2) three. Unordered.*
15. *(‡176) P_3_, posterior margin: (0) wide; (1) narrow.*
16. *(‡178) P_3_, posterior lingual cusp: (0) prominent; (1) weak or absent.*
17. *(‡179) P_3_, cingulum: (0) present, complete; (1) present, partial; (2) absent. Unordered.*
18. *(‡177) P_3_, metaconid crest: (0) present; (1) absent.*
19. *(‡178) P_3_, posterolingual cusp: (0) present; (1) absent.*
20. (‡32) P^3^; (0) present; (1) absent.
21. (#58) P^3^, lingual lobe (=protocone); (0) present, well developed; (1) vestigial or absent. (Note, See Gould [2] TS†73 for an expanded discussion on this TS.)
22. (#59) P^3^, size; (0) normal; (1) reduced.
23. (†76) P^3^, hypocone; (0) present; (1) absent. This TS is uninformative.
24. (#61) P_4_, shape: (0) with elongated talonid and talonid basin; (1) with a short bicuspid or unicuspid heel. (Note: see Gould [2], TS†77 for more comments about this TS). This TS is uninformative.
25. (‡181) P_4_, talonid size: (0) constitutes greatest breadth of tooth; (1) not.
26. (#61) P^4^, shape and hypocone: (0) quadrate, hypocone present; (1) triangular, hypocone absent or vestigial. (Note: see Gould [2], TS†78 for more comments about this TS.)
27. (‡54) P^4^, hypocone gross size: (0) smaller than the protocone; (1) equal to or greater *than the protocone.*
28. (#63) Trigonid height: (0) high, talonid short, or vestigial; (1) low trigonid, talonid expanded, large.
29. (†81) M_1_, morphology: (0) trigonid moderate; (1) marked elongation of prevallid shear on M_1_.
30. (‡208) M_1_, hypoconid: (0) isolated from other cusps; (1) attached to other cusps by a crest; (2) absent. Unordered.
31. (‡209) M_1_, entoconid height: (0) greater than the hypoconid; (1) greater than the protoconid; (2) approximately equal to all cusps; (3) greater than both cusps; (4) smallest cusp. Unordered.
32. (‡221) M_1_, paracristid: (0) terminates at the midanterior of the ridge, does not reach protocone; (1) extends to protocone.
33. (‡69) M^1^, shape: (0) transversely rectangle; (1) antero-posteriorly rectangle; (2) quadrate. Unordered. This TS is uninformative.
34. (#64) M^1^, lingual roots: (0) separate; (1) fused for most of the length.
35. (‡72) M^1^, metaconule: (0) present; (1) absent.
36. (‡78) M^1^, protocone position: (0) anterior to the paracone; (1) posterior to the paracone; (2) approximately equivalent with the paracone. Unordered.
37. (‡88) M^1^, hypocone height: (0) tallest cusp; (1) shortest cusp; (2) approximately equivalent in height with other cusps. Unordered.
38. (‡93) M^1^, hypocone position: (0) isolated from all other cusps; (1) connected to other cusps by a crest.
39. (‡95) M^1^, metastyle apex: (0) high; (1) low; (2) absent. Unordered.
40. *(‡235) M_2_, hypoconid positions: (0) lingual to protocone; (1) labial to protoconid; (2) approximately equally aligned. Unordered.*
41. *(‡238) M_2_, talonid basin: (0) enclosed lingually; (1) not.*
42. *(‡241) M_2_, entoconid cristid: (0) high; (1) low; (2) absent. Unordered.*
43. *(‡236) M_2_, entoconid size: (0) equal to protoconid; (1) greater than paracone; (2) smaller than paracone. Unordered.*
44. *(‡106) M^2^, lingual roots: (0) fused; (1) separate; (2) one. Unordered.*
45. *(‡109) M^2^, protocone height: (0) equal to paracone; (1) greater than paracone; (2) smaller than paracone. Unordered.*
46. *(‡111) M^2^: (0) isolated from all other cusps; (1) attached to other cusps by a crista.*
47. (†84) M^3^; (0) present; (1) absent. This TS is uninformative.
48. (‡133) M^3^; protocone size: (0) large; (1) small.
49. (#66) M^3^, metacone; (0) present; (1) absent. (Note, See Gould [2], TS†86 for an expanded discussion on this TS.)
50. (‡243) M_3_: (0) present; (1) absent.
51. (‡248) M_3_, trigonids: (0) high; (1) short, (2) low, expanded.
52. (‡245) M_3_, paraconid shelf: (0) present; (1) absent.
53. (#68) M_3_, talonid: (0) present; (1) absent.
54. *(new) Diastema between teeth: (0) present; (1) absent.* Added by Gould [4] to accommodate several fossil taxa.
55. *(new) Diastema between I^3^ and upper canines: (0) present; (1) absent. Added by Gould [4] to accommodate several fossil taxa.*
56. *(new) Diastema between upper canine and succeeding premolar: (0) present; (1) absent. Added by Gould [4]to accommodate several fossil taxa.*
57. *(new) Diastema between first two upper premolars: (0) present; (1) absent. Added by Gould [4] to accommodate several fossil taxa.*
58. *(new) Diastema between lower canine and I_3_: (0) present; (1) absent. Added by Gould [4] to accommodate several fossil taxa.*
59. *(new) Diastema between lower canine and succeeding premolar: (0) present; (1) absent. Added by Gould [4] to accommodate several fossil taxa.*

**Postcranial Characters:**

1. (#69) Axis, postventral keel; (0) present; (1) absent.
2. (#70) Axis, neural spine; (0) low; (1) high.
3. (#71) Scapula, metacromion process: (0) deltoid, amorphous projection; (1) long, fusiform projection.
4. (#73) Sacral vertebrae, neural spines: (0) not fused into a continuous longitudinal plate; (1) fused into a continuous longitudinal plate.
5. (#73) Ischium, posterdorsal process; (0) not greatly elongated; (1) greatly elongated.
6. (#74) Tibia, lateral flange: (0) absent or only weakly present; (1) lateral flange on anterosuperior margin strongly developed.

**Pelage Characters:**

1. (#75) External pinnae, length relative to condlyoincisive length: (0) short; (1) long.
2. (#76) Hallux: (0) normal; (1) reduced, claw not reaching base of second digit; (2) absent.
3. (†97) Pelage spines: (0) absent; (1) present.
4. (#77) Pelage condition; (0) smooth, spines; (1) spines papillate, not grooved; (2) spines papillate and grooved. Unordered.
5. (#78) Pelage, completely white spines among dark: (0) absent; (1) present. Unordered.
6. (†100) Spines on top of head: (0) absent; (1) present.
7. (#79) Pelage, medial tract on crown: (0) spineless, medial crown tract narrow; (1) spineless, medial crown tract wide; (2) spines papillate and grooved; (3) spineless, medial crown tract absent. Unordered. (Note, See Gould [2] TS†101 for an expanded discussion on this TS.)
8. (#80) Pelage condition on ventral side; (0) soft and densely furred; (1) coarse and relatively sparsely haired.
9. (#81) Pelage, body under fur: (0) absent; (1) present.

**References:**

1. Frost DR, Wozencraft WC, Hoffmann RS (1991) Phylogenetic relationships of hedgehogs and gymnures (Mammalia, Insectivora, Erinaceidae): Smithsonian Contributions to Zoology. 69 p.

2. Gould GC (1995) Hedgehog phylogeny (Mammalia, Erinaceidae): The reciprocal illumination of the quick and the dead. American Museum Novitates 0: 1-45.

3. Gould GC (2001) The phylogenetic resolving power of discrete dental morphology among extant hedgehogs and the implications for their fossil record. American Museum Novitates: 1-52.

4. Gould GC (1997) Systematic revision of the Erinaceidae (Mammalia)-a comprehensive phylogeny based on the morphology of all known taxa. New York: Columbia University.
